# Major vessel occlusion may predict subtherapeutic anticoagulation intensity and feasibility of administration of intravenous thrombolytics

**DOI:** 10.1371/journal.pone.0170978

**Published:** 2017-02-03

**Authors:** Jun Young Chang, Seunguk Jung, Hyun Park, Moon-Ku Han

**Affiliations:** 1 Department of Neurology, Gyeongsang National University Changwon Hospital, Changwon, Republic of Korea; 2 Department of Neurosurgery, Gyeongsang National University Changwon Hospital, Changwon, Republic of Korea; 3 Department of Neurology, Seoul National University Bun-Dang Hospital, Seongnam, Republic of Korea; "INSERM", FRANCE

## Abstract

**Objective:**

We investigated the association between the presence of major vessel occlusion (MVO) and the intensity of the International Normalized Ratio (INR) in cardioembolic high-risk patients taking warfarin. We also evaluated whether the presence of MVO could predict the subtherapeutic range of INR ≤1.7 ensuring safe administration of intravenous thrombolytics.

**Methods:**

The medical records of 177 cardioembolic stroke patients who were taking warfarin between April, 2008 and March, 2015 were retrospectively analyzed. Logistic regression analysis was performed to calculate the odds ratios (ORs) and 95% confidence intervals (95% CIs) for the association between vessel occlusion and intensity of INR. To predict INR ≤1.7, decision tree analysis was performed.

**Results:**

INR was inversely associated with MVO in an unadjusted model (OR, 0.36; 95% CI, 0.17–0.76), and in a model adjusted for initial NIHSS score and time from symptom onset to arrival (OR, 0.28; 95% CI, 0.11–0.73). Fifty-two of 58 (89.7%) patients with MVO had an INR ≤1.7, compared with 83 of 119 (69.7%) patients without MVO. Indication for anticoagulation agent use was dichotomized into NVAF and others, and applied to the subgroup of patients with MVO. All patients with NVAF (31/31, 100%) had INR ≤1.7, while 21 of 27 of the other patients (77.8%) had INR ≤1.7.

**Conclusions:**

Low INR at presentation in cardioembolic stroke patients during anticoagulation treatment was associated with occurrence of major vessel occlusive stroke. Presence of MVO and indications for anticoagulation may be utilized to ensure the feasibility of administration of intravenous thrombolytics.

## Introduction

Cardioembolic stroke is a frequent event, constituting 14 to 30% of cases of acute ischemic stroke, and is the most lethal of stroke subtypes. [1, 2] Because of the increased prevalence of atrial fibrillation in the elderly population, warfarin use increased from 29% to 63% from 1992 to 2010. [3] Though anticoagulation therapy effectively reduces stroke recurrence by two-thirds and mortality by one-quarter in patients with nonvalvular atrial fibrillation, [4] long-term maintenance of an International Normalized Ratio (INR) within a therapeutic range as well as adherence to a warfarin regimen is difficult to attain. INR was outside the target range 32.1% of the time in warfarin-medicated patients, half of whom had INR <2.0. [5] Moreover, about one- quarter of atrial fibrillation patients who were prescribed warfarin quit taking the drug within 1 year. [6] As a result, the number of acute ischemic stroke patients taking oral anticoagulants is increasing in emergency departments. Current guidelines indicate the use of intravenous thrombolytic agents in patients with INR levels equal to or below 1.7 to avoid the risk of hemorrhagic complications. [7] When immediate thrombolysis is indicated, confirmation of coagulation-related laboratory results must be obtained in patients taking warfarin, which often delays the administration of recombinant tissue plasminogen activator (rtPA). No previous study has attempted to predict the subtherapeutic range of INR in patients with high cardioemobolic risk factors using oral anticoagulation agents. Knowledge of the relevant variables may be helpful in ensuring safe administration of intravenous thrombolytics.

Major vessel occlusion (MVO) is associated with poor 6-month outcomes compared with cases of ischemic stroke without large vessel occlusion. [8] The intensity of INR is correlated with good clinical outcomes, the mechanism of which is presumed to involve formation of smaller thrombi in patients with greater INR intensity. [9] We hypothesized that the initial intensity of INR in acute ischemic stroke patients with cardioembolic high risk factors on anticoagulation is inversely associated with the presence of MVO. The purpose of this study is to investigate the association between MVO and the intensity of INR in the study population. We also evaluated whether the presence of MVO could predict the subtherapeutic range of INR which would ensure safe administration of intravenous thrombolytics.

## Methods

We retrospectively reviewed the medical records of the patients from a single hospital-based stroke registry. Patients with cardioembolic stroke taking warfarin who visited the institute within 24 hours from symptom onset between April, 2008 and March, 2015 were included in the analysis. Time from symptom onset was based on the first time the patient reported feeling abnormal or having symptoms. Cardioembolism was assigned when ischemic lesion were due to embolus of cardiac origin without any other embolic sources or significant stenosis at the relevant cerebral artery on angiographic studies. According to the TOAST classification, cardioembolic high risk includes mechanical prosthetic valve, mitral stenosis with atrial fibrillation, atrial fibrillation (other than lone atrial fibrillation), left atrial/atrial appendage thrombus, sick sinus syndrome, recent myocardial infarction (<4 weeks), left ventricular thrombus, dilated cardiomyopathy (ejection fraction <35%), akinetic left ventricular segment. [10] Besides the mentioned above, patients with patent foramen ovale (PFO) and a venous source of embolism, rheumatic mitral valve disease, atrial flutter were also indicated for anticoagulation and included in the analysis. Baseline demographics (age, sex), clinical characteristics (INR at presentation, initial NIHSS score, time from symptom onset to arrival, vascular risk factors, and cardioembolic risk factors) were collected from the registry. Institutional Review Board (IRB) of the study hospital approved the retrospective study.

The initial INR value taken in the emergency room visit was used for the study. Vessel occlusion is defined as complete signal loss distal to the occlusion site on CT angiography (CTA) or magnetic resonance angiography (MRA). Vessel occlusion sites were categorized into the internal carotid artery (ICA), the middle cerebral artery (MCA) (M1 and M2 segments), the basilar artery (BA), the posterior cerebral artery (PCA) (P1 segment), the anterior cerebral artery (ACA) (A1 segment), and the vertebral artery (VA). Angiographic data were read by an experienced neuroradiologist and reviewed by two neurologists. Occlusion site were established by 2-reader consensus (C.J.Y, J.S.W). Functional outcome after 3 months was determined by a neurologist at the outpatient clinic, or through a structured telephone interview with a trained nurse. A favorable outcome was defined as a Modified Rankin Scale (MRS) score ≤2.

### Statistical analysis

Baseline demographics and clinical characteristics were compared according to the presence of major vessel occlusion. Chi-square or Fisher’s exact tests were used to analyze categorical variables, while independent *t* tests or Mann-Whitney *U* tests were used for continuous variables. Logistic regression analysis was performed to calculate the odds ratios (ORs) and 95% confidence intervals (95% CIs) for the association between vessel occlusion and intensity of INR (unadjusted, adjusted for initial NIHSS score, or adjusted for initial NIHSS score and time from symptom onset to arrival). Area under the receiver operating characteristic (ROC) curve was compared to evaluate the predictive power of the model. Sensitivity analysis was performed that included patients with nonvalvular atrial fibrillation (AF). The linearity assumption for continuous variables was evaluated using restricted cubic splines. Goodness of fit was evaluated using the Hosmer-Lemeshow test. The predictive logistic model was internally validated by bootstrap simulations. These analyses were performed using Stata version 13.0 (Stata Corp., Texas, USA).

To predict INR ≤1.7 for safe administration of intravenous rtPA, we performed decision tree analysis using R version 3.2.2 (R: A language and environment for statistical computing. R Foundation for Statistical Computing, Vienna, Austria). Decision tree analysis was developed using the Classification Tree method. Specifically, conditional inference tree algorithm was used. A Chi-square test was used to explore the association between binary outcomes and candidate variables, and the most significant variable was selected for splitting. The permutation test framework was used for optimal binary splitting of the selected variable. [11] All tests were 2-sided, and P values <0.05 were considered statistically significant.

## Results

Of the 3,276 ischemic stroke patients who visited the institute within 24 hours of symptom onset during the study period, 951 (29%) were diagnosed with cardioembolic stroke according to the MAGIC algorithm. [12] Among them, 177 (5.4%) patients at high risk for cardioembolic stroke who were taking warfarin before the event fulfilled the inclusion criteria and were finally included in the analysis. The mean age of the study subjects was 73.2 years, and 48% were male. Thirty-two (18.1%) patients represented wake up stroke. The median from symptom onset to arrival based on last seen normal time was 218 minutes (interquartile range, 61–630). The median from symptom onset to arrival based on first abnormal time was 103 minutes (interquartile range, 45–276). The median baseline NIHSS score was 8 (interquartile range, 3–15), and hypertension (72.3%) was the most frequent risk factor. Major vessel occlusion was noted in 58 (32.8%) of the study subjects. Vessel occlusion site was as follows in order of frequency; M1 (20, 34.5%), M2 (15, 25.9%), intracranial ICA (12, 20.7%), extracranial ICA (3, 5.2%), ACA (2, 3.4%), BA (2, 3.4%), PCA (1, 1.7%), and VA (1, 1.7%). Nonvalvular atrial fibrillation (100, 56.5%) was the most frequent cause of anticoagulation among the study population, followed by vavular atrial fibrillation, and left atrial appendage thrombus (Table 1).

**Table 1:** Causes of anticoagulation.

Cause, n (%)	
NVAF	100 (56.5)
VAF	56 (31.6)
LAA thrombus	5 (2.8)
LV akinesia without thrombus	4 (2.3)
Recent MI	3 (1.7)
Mechanical prosthetic valve	2 (1.1)
Atrial flutter	2 (1.1)
LV thrombus	2 (1.1)
PFO	1 (0.6)
CHF	1 (0.6)
Rheumatic MV prolapse	1 (0.6)

Abbreviations: NVAF, nonvavular atrial fibrillation; VAF, valvular atrial fibrillation; LAA, left atrial appendage; LV, left ventricle; MI, myocardial infarction; PFO, patent foramen ovale; CHF, congestive heart failure; MV, mitral valve.

Baseline demographics and clinical characteristics according to presence of major vessel occlusion are listed in Table 2. The median INR value in patients with MVO was 1.2 (interquartile range, 1.1–1.6), which was significantly lower than the median value of 1.3 (interquartile range, 1.1–1.9) seen in patients without vessel occlusion. The proportion of patients with INR >1.7 and ≥2.0 were significantly higher amongst those without MVO. The severity of stroke was significantly greater, and time from symptom onset to arrival was shorter in the patients with MVO. No significant difference was observed in the frequency of vascular risk factors, cardioembolic risk factors dichotomized into nonvalvular atrial fibrillation (NVAF) and others, and laboratory findings including total cholesterol, serum glucose, and HbA1c. The rate of favorable functional outcomes at 90 days tended be higher in patients without MVO but was not significantly different between the two groups (Table 2).

**Table 2:** Comparison of baseline characteristics and variables according to presence of major vessel occlusion.

	Total (n = 177)	Major vessel occlusion		p value
		Major vessel occlusion(+) (n = 58)	Major vessel occlusion(-) (n = 119)	
Age,y				
Mean (SD)	73.2 (10.7)	73.8 (10.2)	73.0 (10.9)	
Median (IQR)	74.0 (69.0–80.5)	75 (70–80)	74.0 (69.0–80.0)	0.8
Male, n (%)	85 (48.0)	29 (50.0)	56 (47.1)	0.84
INR†				
Mean (SD)	1.5 (0.7)	1.3 (0.3)	1.6 (0.8)	
Median (IQR)	1.3 (1.1–1.7)	1.2 (1.1–1.6)	1.3 (1.1–1.9)	0.03
INR>1.7, n (%)	42 (23.7)	6 (10.3)	36 (30.3)	<0.01
INR≥2.0, n (%)	28 (15.8)	3 (5.2)	25 (21.0)	0.01
Initial NIHSS†				
Mean (SD)	10.1 (8.2)	14.7 (7.3)	7.8 (7.7)	
Median (IQR)	8.0 (3.0–15.0)	15.0 (10.0–20.0)	5.0 (2.0–12.0)	<0.01
NIHSS≥15	54 (30.5)	32 (55.2)	22 (18.5)	<0.01
Symptom onset to arrival time, min†				
Mean (SD)	226.9 (291.8)	111.7 (131.0)	283.0 (330.2)	
Median (IQR)	103.0 (45.0–276.0)	57.5 (32.0–119.0)	132.0 (60.0–390.0)	<0.01
Risk factors, n (%)				
HT	128 (72.3)	39 (67.2)	89 (74.8)	0.38
DM	49 (27.7)	14 (24.1)	35 (29.4)	0.58
Dyslipidemia	60 (33.9)	23 (39.7)	37 (31.1)	0.34
Smoking	59 (33.3)	16 (27.6)	43 (36.1)	0.34
Previous stroke	87 (49.2)	23 (39.7)	64 (53.8)	0.11
Cardioembolic high risk, n (%)				
NVAF	100 (56.5)	31 (53.4)	69 (58.0)	0.68
Others	77 (43.5)	27 (46.6)	50 (42.0)	
Laboratory findings, median (IQR)				
T.chol	155.0 (134.5–174.5)	154.0 (137.0–186.0)	155.0 (129.0–171.0)	0.19
Glucose	125.0 (104.0–153.0)	122.5 (109.0–142.0)	126.0 (103.5–158.5)	0.65
HbA1c	5.9 (5.5–6.4)	5.9 (5.7–6.3)	5.9 (5.5–6.5)	0.68
MRS≤2 at 3month, n(%)	81 (45.8)	22 (37.9)	59 (49.6)	0.19

Abbreviations: INR, international normalized ratio; HT, hypertension; DM, diabetes mellitus; NVAF, nonvavular atrial fibrillation; T.chol, total cholesterol; mRS, modified Rankin Scale.

**Fig 1:**
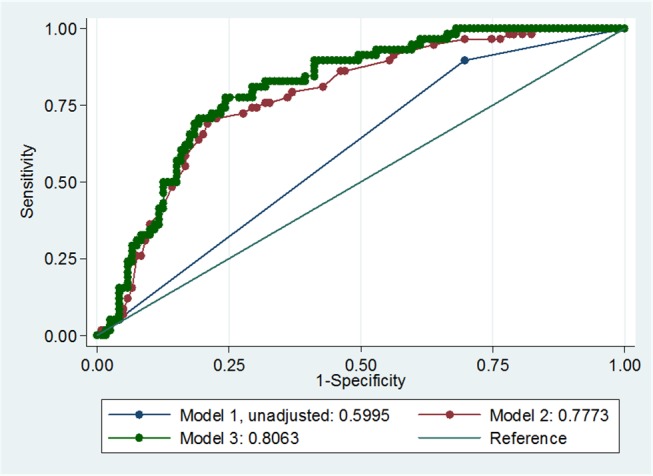
The intensity of INR and MVO- Predictability of the models.

An increase in INR as a continuous variable was inversely associated with MVO in the unadjusted model (unadjusted OR, 0.36; 95% CI, 0.17–0.76). After adjusting for initial NIHSS score and time from symptom onset to arrival, this association remained significant (adjusted OR, 0.28; 95% CI, 0.11–0.73, Table 3). The calculated area under the ROC curve for the unadjusted model, model 2 (adjusted for initial NIHSS score), and model 3 (adjusted for initial NIHSS score and time from symptom onset to arrival) were 0.60, 0.78, and 0.81, respectively (p = <0.01, Fig 1). After dichotomizing the intensity of INR by 1.7 and 2.0, respectively, a higher level of INR was also found to be an independent predictor of the absence of MVO in multivariate analysis (Table 3).

**Table 3:** Association of the intensity of INR and major vessel occlusion.

	Model 1, Unadjusted, Odd ratio (95% Confidence Interval)	Model 2, *Odd ratio (95% Confidence Interval)	Model 3, ¶Odd ratio (95% Confidence Interval)
**INR**	0.36 (0.17–0.76)	0.30 (0.12–0.75)	0.28 (0.11–0.73)
**INR0.8–1.2 (reference)**			
**INR 1.2–2.0**	1.19 (0.55–2.58)	0.85 (0.36–2.02)	1.02 (0.41–2.56)
**INR >2.0**	0.21 (0.06–0.75)	0.20 (0.05–0.78)	0.18 (0.05–0.69)
**INR >1.7**	0.27 (0.10–0.68)	0.27 (0.10–0.74)	0.27 (0.09–0.75)
**INR ≥2.0**	0.21 (0.06–0.71)	0.22 (0.06–0.81)	0.18 (0.05–0.68)

* Adjusted for initial NIHSS.

¶ Adjusted for initial NIHSS and symptom onset to arrival time.

**Fig 2:**
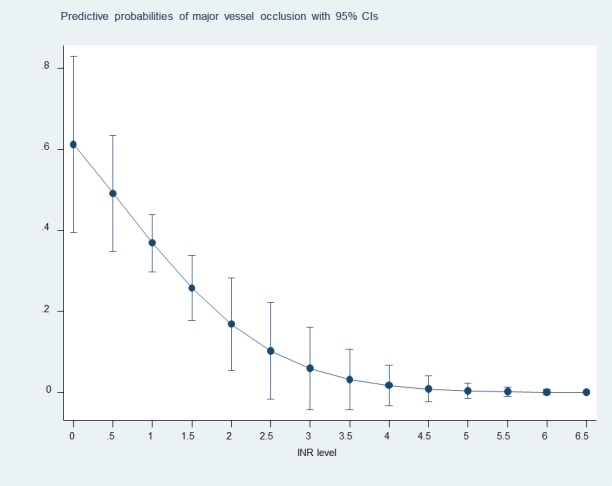
The predicted probability of MVO according to INR level.

The predicted probability of MVO according to INR level is presented in Fig 2.

**Fig 3:**
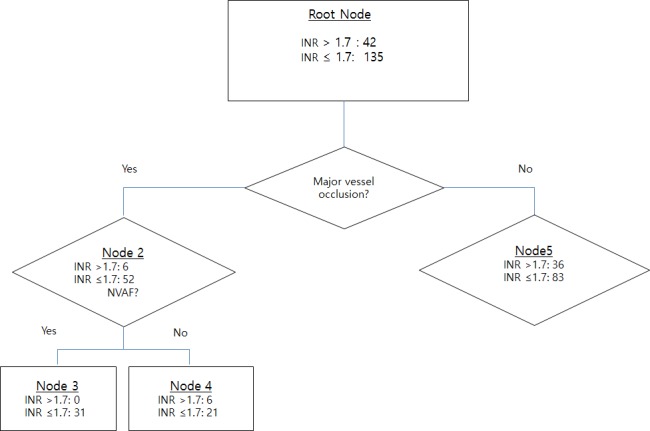
Decision tree analysis employed in the current study for the prediction of safe administration of rtPA.

The initial INR level was below or equal to 1.7 in 135 of 177 (76.3%) patients. Presence of MVO was the best predictor for safe administration of rtPA. Fifty-two of 58 (89.7%) patients with MVO had INR ≤1.7, compared with 83 of 119 (69.7%) patients without MVO. When indication for anticoagulation was dichotomized into NVAF versus others and applied to the subgroup patients with MVO, all patients with NVAF (31/31, 100%) had INR ≤1.7, while 21 of 27 patients with the “other” etiology (77.8%) had INR ≤1.7 (Fig 3).

## Discussion

The results of our study show that the initial level of INR during anticoagulation in high-risk cardioembolic stroke patients is inversely associated with major vessel occlusive stroke. Our study is unique in that, by utilizing factors such as presence of MVO and type of cardioembolic risk factor and conducting analysis by a decision tree method, we attempted to ensure the safety of intravenous thrombolysis. Maintenance of INR ≥2.0 during anticoagulation in patients with NVAF reduces stroke-related mortality as well as stroke recurrence. [13] A study from the Fukuoka Stroke Registry reported that baseline INR ≥2.0 was associated with less severe initial neurological deficits and favorable functional outcomes at discharge. [9] The mechanism of this result was assumed to be the consequence of smaller, more resolvable thrombi formation, thus leading to rapid, spontaneous recanalization of occluded arteries. [9] However, the vascular status of the subjects was not evaluated in the study. Intracranial large vessel occlusion in acute ischemic stroke was significantly associated with severe baseline NIHSS score and independent prognostic factors for poor outcomes. [14] The inverse correlation between intensity of INR and MVO demonstrated in our study could be the reason why preadmission INR ≥2.0 was more likely to cause stroke of reduced severity and lead to good outcomes. MVO represents not only an outcome predictor, but also an indicator for the need for urgent transfer to a higher care center for revascularization.

In this era of endovascular recanalization, intravenous thrombolysis should still be performed as early as possible, within 4.5 hr of symptom onset, with earlier administration improving functional outcomes at 3 to 6 months. [15] According to the current guidelines, clinicians should do our best to reduce any time delay in rtPA administration. [16] However, to avoid hemorrhagic complications related to rtPA, confirmation of INR level ≤ 1.7 should be done in patients already taking vitamin K antagonists, which inevitably results in a time delay to thrombolytic administration. Though utilization of point of care (POC) testing could be an alternative method of reducing the waiting time for an INR report with acceptable validity, [17] universal access to such information still has a long way to go. A precise but simple way of predicting subtherapeutic INR with clinical variables is therefore worthy of investigation.

[18] Intravenous thrombolysis is allowed in warfarin-treated patients only in cases with INR ≤ 1.7, which has been shown to be safe in several observational studies and is documented in the current guidelines. [7, 19] The presence of MVO was found to be the best predictor of final outcomes in our study, with its presence associated with an increased possibility of achieving INR ≤ 1.7. The median values of door to first image time and door to laboratory report time were 26 min (interquartile range, 21–34) and 40 (interquartile range, 31–53), respectively, in our institute (data not shown). Utilizing angiographic information before obtaining time-consuming coagulation test results could be helpful in reducing door-to-rtPA time.

The addition of ‘indication for anticoagulation’ at the second level of the decision tree further refined the prediction of INR ≤1.7. Subtherapeutic INR is frequently encountered in clinical practice, ranging from 25% to 34% of cases according to definition and study population. [18, 20] Only 38% of patients with atrial fibrillation were reported to achieve INR ≥2.0 according to a Japanese registry. [9]Indications for anticoagulation have an influence on INR level, with venous thromboembolism more likely to be associated with failure to achieve a therapeutic range of INR and presence of a mechanical heart valve less likely to lead to low INR. [18] Different ranges of target INR may be attributable to different proportions of subtherapeutic INR according to indications for anticoagulation. Patients with mechanical mitral valves may be less likely to experience INR ≤1.7 because of the higher target INR of 3.0 and more likely to be on vigilant monitoring of INR level.

By combining the two variables, ‘presence of MVO’ and ‘indication of anticoagulation,’ we can infer that large vessel occlusion rarely occurs from clots originating from the heart in patients with NVAF once the INR level is maintained above 1.7, whereas intracerebral artery occlusion could occur by the formation of cardiac emboli in patients with other high cardioembolic risk factors except NVAF even if their INR level is above 1.7. According to underlying cardiac disorders, size of cardiac emboli and thromboresistance may differ between such patient populations. [21] Compared with a clot arising from the left atrial appendage in patients with NVAF, left ventricular (LV) thrombus in patients with recent myocardial infarction or severe heart failure may be larger. Compared with patients with NVAF, a higher INR target is required for patients with mechanical prosthetic mitral valves, which indicates more thrombogenic conditions despite anticoagulation.

This study has several limitations. First, the decision tree model developed based on the small number of retrospective study subjects needs further validation in a multicenter, prospective cohort to improve its predictive power. Second, various reasons for the subtherapeutic range of INR, such as warfarin nonadherence and duration of warfarin interruption were not investigated. These factors could also be utilized as an important predictor for initial intensity of INR. Third, the INR taken after emergency room visit might not represent the INR at the onset of stroke. However INR value was significantly lower even though symptom onset to arrival time was shorter in patients with MVO. It is less likely that the difference of INR value was merely due to time difference of symptom onset to arrival between the two groups.

## Conclusions

Low INR in patients with anticoagulation was associated with occurrence of major vessel occlusive stroke. The presence of MVO and indications for anticoagulation may be utilized as clues to predict low INR in an effort to ensure the feasibility of administration of intravenous thrombolytics. However, the results of this study should be interpreted cautiously and regarded as hypothesis generating study which needs further prospective confirmation.

## Supporting information

S1 Fig Flow diagram of the study subjects. (TIF)
