# Supplementary figures and images for: Major vessel occlusion may predict subtherapeutic anticoagulation intensity and feasibility of administration of intravenous thrombolytics
Source: PLoS One. 2017 Feb 3;12(2):e0170978. doi: 10.1371/journal.pone.0170978 (PMC5291417; doi:10.1371/journal.pone.0170978)

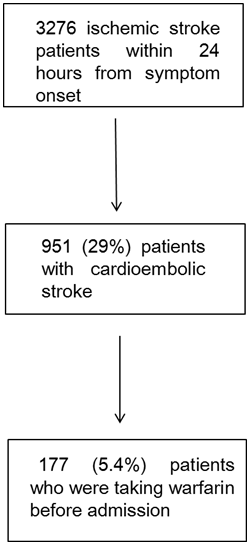

Supplement: S1 Fig — (TIF) [file pone.0170978.s001.tif]
